# Therapeutics for Dengue: Recommendations for Design and Conduct of Early-Phase Clinical Trials

**DOI:** 10.1371/journal.pntd.0001752

**Published:** 2012-09-27

**Authors:** Cameron P. Simmons, Marcel Wolbers, Minh Nguyet Nguyen, Jamie Whitehorn, Pei Yong Shi, Paul Young, Rosemary Petric, Van Vinh Chau Nguyen, Jeremy Farrar, Bridget Wills

**Affiliations:** 1 Oxford University Clinical Research Unit, Hospital for Tropical Diseases, Ho Chi Minh City, Vietnam; 2 Centre for Tropical Medicine, Nuffield Department of Medicine, University of Oxford, Oxford, United Kingdom; 3 Hospital for Tropical Diseases, Ho Chi Minh City, Vietnam; 4 Hoffmann-La Roche, Nutley, New Jersey, United States of America; 5 Novartis Institute for Tropical Diseases, Biopolis Way, Singapore; 6 London School of Hygiene and Tropical Medicine, London, United Kingdom; 7 School of Molecular Biosciences, University of Queensland, Brisbane, Queensland, Australia; The George Washington University Medical Center, United States of America

## A Standardized Approach to Early-Phase, Proof-of-Concept Therapeutic Trials in Dengue

This Perspective article reflects discussions held at the 1^st^ International Dengue Therapeutics Workshop, held in Ho Chi Minh City, Vietnam in September 2011, attended by participants from academia and industry, and focused on recent experience from several randomized controlled clinical trials of candidate dengue therapeutic agents [1], [2], [3]. The aim of this article is to provide an informed set of recommendations for the design and conduct of early-phase clinical trials where safety and antiviral efficacy are the endpoints of interest. The focus for this article is on early-phase testing of antiviral therapies because this is where most drug development efforts in academia and industry are directed. Establishing a standardized and consistent framework for dengue therapeutic trials will facilitate better data management and analytical practices and ensure that clinical data can be readily collated and made available for pooled analyses.

## The Rationale for Dengue Therapeutics

Dengue is a self-limiting, systemic illness caused by any of the four dengue viruses, DENV-1 to DENV-4, and usually resolves without complications after 3–7 days. However, in some patients a transient vasculopathy develops between days 3–6 of illness. Marked increases in vascular permeability may occur in some cases of sufficient severity to precipitate life-threatening hypovolemic shock—i.e. dengue shock syndrome (DSS). The increased permeability is often accompanied by thrombocytopenia and a bleeding diathesis that together may result in frank hemorrhage from mucosal sites. Currently, there are no specific therapies for treating dengue and management consists of supportive care only [4], [5]. Against this backdrop, development of a therapeutic strategy that attenuates the duration and severity of symptoms and/or reduces the incidence or magnitude of these major complications has become a priority. An effective antiviral therapy could result not only in fewer patients requiring admission to hospital, but could potentially also reduce transmission of dengue viruses to mosquitoes. In recognition of these possible benefits, there is an expanding effort in academia and industry to identify and develop novel therapeutics for dengue.

## Considerations in Clinical Trial Design and Conduct

### Patient Populations and Baseline Features

Early-phase trials of a novel therapy usually limit enrollment to young, otherwise healthy adults in order to gather the most “noise free” profile of drug safety. However in high transmission settings, dengue is primarily a disease of children and young adults, with DSS the commonest severe complication seen in pediatric dengue patients. Therefore, as favorable safety and antiviral efficacy data accumulate in adults, clinical development would need to progress to include pediatric age groups where the incidence of severe dengue is highest.

### Diagnosis

Prompt and rapid diagnosis of dengue for potential trial participants is possible using NS1 rapid tests [6] or RT-PCR. DENV plasma viremia (probably a reasonable surrogate marker of the underlying systemic infection) typically peaks within 24–48 h of fever onset and declines rapidly over the ensuing days such that the majority of patients have resolved their viremia and are afebrile 5–7 days after symptom onset [7],[8],[9]. Thus ideally diagnosis, screening, and enrollment; randomization; and commencement of treatment with a candidate antiviral drug should all occur within 24–48 h of fever onset.

### Deciphering Drug Safety Signals from the Natural History of Dengue

**Figure 1:**
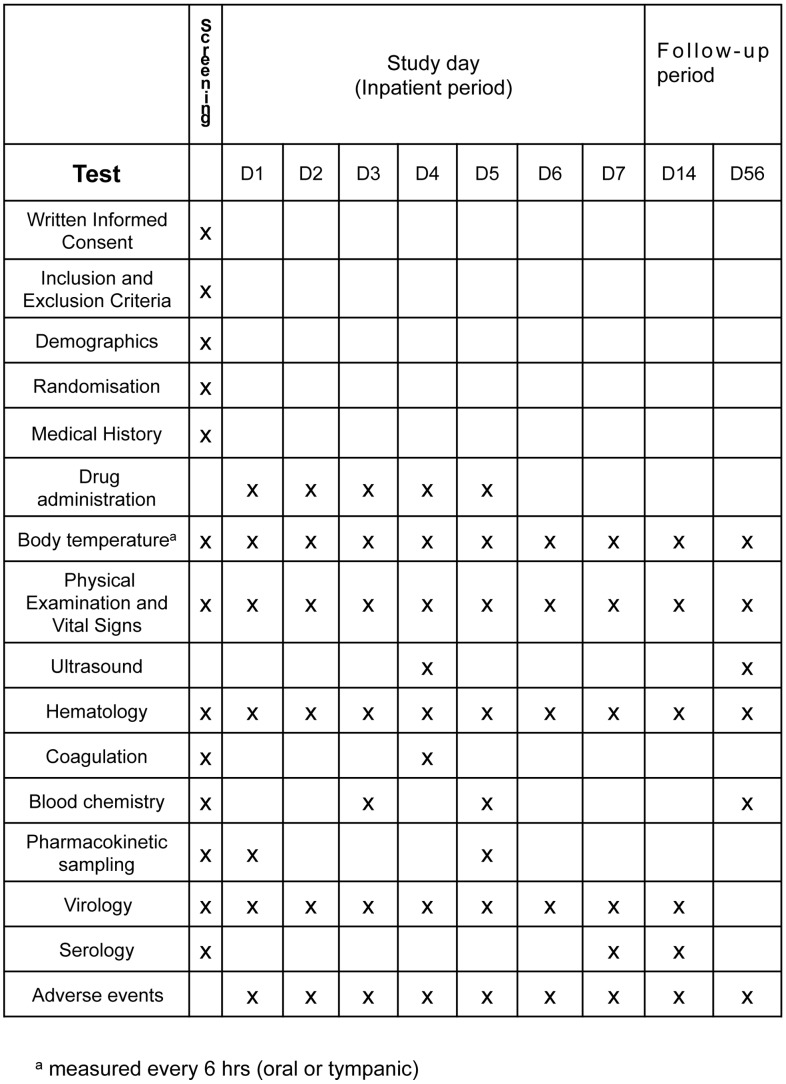
Suggested minimum schedule of assessments for a trial of a candidate therapeutic in a patient population with <48 h of fever at the time of commencing treatment.

The natural history of clinical dengue evolves through several different stages and involves a range of clinical and laboratory changes, some common and usually minor (e.g. skin petechiae and thrombocytopenia) and others rare but clinically significant (e.g. encephalopathy or transient loss of visual acuity) [4], [5]. Distinguishing between adverse events that are manifestations of dengue as opposed to drug-safety signals relies on informed clinical judgment; invariably the best clinical judgment will be found amongst clinicians who routinely manage dengue cases in endemic settings. Thus we recommend that participants in early-phase trials should be managed as inpatients for 5–7 study days. Follow-up visits at days 14 and 56 should be sufficient to observe resolution of the recognized dengue-related clinical and laboratory adverse events and to facilitate collection of important “baseline” laboratory data for each patient (see Figure 1).

### Clinical and Routine Laboratory Endpoints

During the inpatient period, routine monitoring of body temperature and vital signs should be conducted at least 6-hourly and complemented with daily physical examination and assessment of symptoms. Routine hematological and biochemical investigations should include the following: daily complete blood count; serial measurements of liver and renal function; and coagulation profiles at enrollment, during the critical phase for complications (day 4–6 of illness), and in convalescence. Additional investigations may be indicated depending on the known side-effect profile of the drug under investigation. All tests should be repeated at follow-up to establish “baseline” values for each patient. Reporting of laboratory tests should focus on measures to assess the severity of vascular leakage (e.g. overall percentage change in haematocrit with respect to the baseline for that individual) as well as thrombocytopenia, the coagulation profile, and hepatic transaminase levels—all of which provide useful, continuous measures of disease evolution and severity. Daily ultrasound to detect signs of capillary leakage during the critical phase should also be considered, and ideally performed by a single experienced sonographer to minimize interobserver variability. The schedule of recommended clinical and laboratory investigations is shown in Figure 1. To allow for comparative analyses between studies, the recommended methods of reporting these variables are shown in Table 1. Although one of the aims of therapeutic-drug development is to prevent severe complications such as DSS from occurring, it is unlikely that early-phase trials in adults will be powered to detect such an effect given the low incidence of severe dengue in adults. Movement into pediatric dengue case populations will typically occur via age de-escalation studies after acceptable safety and drug activity data has been acquired in adults. Although the frequency of severe complications in pediatric dengue cases (∼4% of cases presenting to primary health care) is higher than in adults, large sample sizes would nonetheless still be required to demonstrate clinical efficacy in preventing severe dengue.

**Table 1:** Standard analyses and reporting of clinical and virological endpoints in early-phase trials of dengue therapeutics.

Variable*	Endpoint to report for each treatment arm
Adverse events	Listings and summaries of adverse and severe adverse events with reference to standard definitions (e.g. Common Terminology Criteria for Adverse Events (CTCAE) v4.0 (Cancer Therapy Evaluation Program, NIH)).
Fever	Fever clearance time—defined as the time from the start of treatment to the start of the first 24-h period during which the tympanic or oral temperature remains below 37.5°C (with 6-hourly measurements).
Hemorrhage	Proportions of patients with mild, moderate, or severe bleeding according to the following definitions:MILD: petechiae and/or minor self-limiting mucosal bleeding that does not require an intervention (e.g. minor nose bleeds, gum bleeds)MODERATE: by clinical judgement, more severe skin or mucosal bleeding but not requiring a transfusion or fluid resuscitation (e.g. small haematemesis or widespread bruising/ecchymoses)SEVERE: bleeding causing haemodynamic compromise requiring fluid resuscitation or use of blood products; any intracranial bleed (regardless of need for fluids); any bleed causing death
Virological features	Area under the log-transformed viremia curve from first dose to the end of study day 7 (AUC)^a^Time to resolution of viremia (<1000 copies/ml)^a^Time to resolution of plasma NS1 antigenemia—defined as the first time NS1 becomes undetectable in plasma
Capillary permeability	Maximum % hemoconcentration—determined by comparison of the maximum Hct detected in the acute phase and a baseline measurement or an age/gender matched population value
Hematology	Minimum platelet count during the critical phaseMaximum aPTT during the critical phaseMaximum INR during the critical phaseMinimum fibrinogen during the critical phase
Biochemistry	Highest hepatic transaminase levels (AST/ALT)

* Comparisons of laboratory and virological features between study groups should be adjusted for the baseline value, the day of illness on admission, and serotype/serology (for virological features) to maximize power.

a AUC calculated based on the trapezoidal rule with values below the limit of detection replaced by half of the detection limit.

### Virological Endpoints

Plasma/serum concentrations of DENV RNA and soluble NS1 should be measured in serial samples collected daily during the treatment period and again at follow-up. Infectious virus can also be measured by classical cell culture techniques, but this approach is low-throughput and less amenable to assay validation. The area under the log-transformed viremia curve (viremia AUC) is a convenient summary measure of the viremia in each treatment arm (see Table 1). Survival curve analysis of NS1 persistence should also be reported and can be complemented with quantitative measurements of NS1 changes over time. Exploratory analyses in one study where twice-daily measurements of viremia were undertaken [2] provided little evidence that more frequent than daily measurements of these virological parameters provide greater sensitivity in detecting differences between treatment arms (data not shown). Genome-scale sequencing of the virus is becoming routine [10] and can be used to provide baseline information on the virus population.

### Dosing and Pharmacology

The pharmacological characteristics of the investigational product will determine the frequency of dosing, but the total course of therapy need not be longer than 5 days' duration given the acute nature of dengue. Consideration of an initial loading dose of an antiviral drug is recommended so as to achieve therapeutic levels at a time when the virus-infected cell mass is at its highest. Pharmacokinetic investigations (e.g., C_max_, C_min_, and T_1/2_) during the trial are essential to define the relationship, if one exists, between in vivo drug parameters and laboratory (e.g. platelet nadir) or clinical features (e.g. frequency of DSS) of disease. Such an understanding should allow for further optimization of dose and timing of therapy. Serum and, if possible, intracellular concentrations of the active moiety and any major metabolites should be measured. If it is not possible to measure the drug moiety itself then surrogates of activity should be explored. Examples of investigations to identify correlates of therapy include serial measurement of whole-blood gene expression profiles, the host immune response (e.g. cytokines, T-cell phenotypes), and pharmacogenomics.

### Non-Invasive Physiological Monitoring

Continuous monitoring of physiological parameters such as heart rate variability, oxygen saturation, respiratory rate, and blood pressure appears to have prognostic significance in the critical care setting [11], [12]. Using these techniques as an observational tool within a dengue clinical trial has the potential to provide useful surrogates of both capillary leak and treatment effect, which could be used as endpoints in further trials.

### Sample Sizes in Early-Phase Studies

Early studies evaluating antivirals should be randomized and powered to detect a treatment effect on the viremia AUC, the suggested primary endpoint. To assess sample size requirements for such a study, we simulated hypothetical treatment effects on top of plasma viremia curves from a previous study [3] and used simulation based on bootstrap resampling for our calculations. Based on these simulations, a total of 30 patients (15 per group) are sufficient to detect an intervention effect of 0.5 log10-copies/ml per day higher viremia clearance with 80% power at the two-sided 5% significance level. We consider this effect size, which can be detected with a small trial, a reasonable estimate of what an effective antiviral might achieve.

### Challenges for the Development of Dengue Therapeutics

Successful therapy with an antiviral agent is highly likely to require early treatment in order to bring about antiviral and physiological responses that are clinically beneficial. A successful drug will need to be rapidly bioavailable and have pharmacological properties that deliver rapid and potent antiviral effects in infected tissues. One practical challenge is that many patients with dengue currently do not present to health care providers until they are several days into their illness, by which time their adaptive immune response is already beginning to resolve their infection. In the future, patient behavior could alter with the availability of effective therapy. Early therapy would also be supported by improvements in the sensitivity of rapid diagnostic tests, e.g. those that detect the NS1 protein.

## Summary

This article describes considerations for the design and conduct of therapeutic trials in dengue. We have recommended a minimum set of clinical and laboratory endpoints in the belief that the field will benefit from a standardized reporting approach. Whilst clear challenges exist in developing therapies for dengue, not least the self-limiting nature of the virus infection, it is hoped that the clinical trial template described here will help accelerate the clinical testing of antiviral and other therapeutic candidates.
